# Evolutionary history of glucose-6-phosphatase encoding genes in vertebrate lineages: towards a better understanding of the functions of multiple duplicates

**DOI:** 10.1186/s12864-017-3727-1

**Published:** 2017-05-02

**Authors:** Lucie Marandel, Stéphane Panserat, Elisabeth Plagnes-Juan, Eva Arbenoits, José Luis Soengas, Julien Bobe

**Affiliations:** 1 INRA, UPPA, UMR 1419 Nutrition, Metabolism, Aquaculture, F-64310 Saint Pée sur Nivelle, France; 2 Departamento de Bioloxía Funcional e Ciencias da Saúde, Laboratorio de Fisioloxía Animal, Facultade de Bioloxía, Universidade de Vigo, E-36310 Vigo, Spain; 3 INRA, UR1037 LPGP, Campus de Beaulieu, F-35000 Rennes, France

**Keywords:** Teleosts, Duplicated genes, Sarcopterygii, Actinopterygii, trout, glucose, brain

## Abstract

**Background:**

Glucose-6-phosphate (G6pc) is a key enzyme involved in the regulation of the glucose homeostasis. The present study aims at revisiting and clarifying the evolutionary history of *g6pc* genes in vertebrates.

**Results:**

*g6pc* duplications happened by successive rounds of whole genome duplication that occurred during vertebrate evolution. *g6pc* duplicated before or around Osteichthyes/Chondrichthyes radiation, giving rise to *g6pca* and *g6pcb* as a consequence of the second vertebrate whole genome duplication. *g6pca* was lost after this duplication in Sarcopterygii whereas both *g6pca* and *g6pcb* then duplicated as a consequence of the teleost-specific whole genome duplication. One *g6pca* duplicate was lost after this duplication in teleosts. Similarly one *g6pcb2* duplicate was lost at least in the ancestor of percomorpha. The analysis of the evolution of spatial expression patterns of *g6pc* genes in vertebrates showed that all *g6pc* were mainly expressed in intestine and liver whereas teleost-specific *g6pcb2* genes were mainly and surprisingly expressed in brain and heart. *g6pcb2b*, one gene previously hypothesised to be involved in the glucose intolerant phenotype in trout, was unexpectedly up-regulated (as it was in liver) by carbohydrates in trout telencephalon without showing significant changes in other brain regions. This up-regulation is in striking contrast with expected glucosensing mechanisms suggesting that its positive response to glucose relates to specific unknown processes in this brain area.

**Conclusions:**

Our results suggested that the fixation and the divergence of *g6pc* duplicated genes during vertebrates’ evolution may lead to adaptive novelty and probably to the emergence of novel phenotypes related to glucose homeostasis.

**Electronic supplementary material:**

The online version of this article (doi:10.1186/s12864-017-3727-1) contains supplementary material, which is available to authorized users.

## Background

The glucose-6-phosphatase 1 (G6pc) enzyme catalyses the hydrolysis of glucose-6-phosphate, produced from glycogen or gluconeogenic precursors, into inorganic phosphate and glucose, which are then released in the extracellular compartment. G6pc plays a crucial role in glucose homeostasis by completing the final step of gluconeogenesis and glycogenolysis. The deregulation of *G6pc* expression thus contributes to several physiological pathologies such as glycogen storage disease type 1a (due to a deficiency in G6pc) [1, 2] or type 2 diabete (due to an overexpression of *G6pc*) [3, 4] in mammals. In these animals, this enzyme is encoded by the *G6pc* gene (glucose-6-phosphatase encoding gene, also called *G6pc1* or *G6pase α*) which is mainly expressed in liver, intestinal mucosa, kidney and pancreatic ß-cell [5].

It was recently demonstrated in teleost fish that g6pc is encoded by 2 to 5 duplicated genes depending on the genus/phylogenetic lineage [6]. Since the insightful suggestion of Ohno in the 70’s [7], whole genome duplication (WGD) has been considered to play a major role in the evolution of vertebrates. Indeed, the origin and evolution of new genes following WGD can provide new molecular and cellular functions, and play an important role in phenotypic variability and genome evolution [8, 9]. After WGD, the duplicated genes can encounter different fates: they can be lost or fixed and maintained with three distinct outcomes, i.e.*,* neofunctionalisation, subfunctionalisation, and conservation of function [10]. Thus, gene duplications have played important roles in the evolution of several biological processes and/or functions including reproduction, development of the human brain, behavioural control as well as the metabolic responses of organisms [8, 9]. Such an adaptive novelty was suggested in rainbow trout in which 5 genes encoding the glucose-6-phosphatase were identified [6]. Indeed, two (*g6pcb2* ohnologs) of these five genes are atypically up-regulated by dietary carbohydrates [6] in the liver of this fish but also by glucose or insulin in vitro [11]. Together with an analysis of the global g6pc enzyme activity, these results strongly suggest a role of the *g6pc* duplicated genes in the establishment of the glucose-intolerant phenotype in trout and thus in the modulation of the metabolic response to a high dietary carbohydrate supply [6]. However, but legitimately on account of its central role in the intermediary metabolism, these conclusions focused only on the liver and are restricted to a single species. To better understand how the presence of additional copies of these genes contributes to phenotypic evolution in different organisms, a closer examination of their general distributions and characteristics in different genomes will be informative [12]. In addition, to assess more comprehensively the functional importance of duplicated genes in driving phenotypic diversity in vertebrates, it will be interesting to take an evolutionary perspective, i.e. related to their ancestral genes. In order to conduct such an analysis, the evolutionary history of *g6pc* has to be thoroughly described. Indeed, during vertebrates’ evolution several rounds of whole genome duplication (WGD) occurred [13]: one at the emergence of chondrichthyes (called the 2R), one at the radiation of teleosts (Ts3R, teleost-specific whole genome duplication) and one at the radiation of salmonids (Ss4R, salmonid-specific whole genome duplication). These WGD duplications led to gene duplication. Moreover, duplicated genes can also originate from small-scale duplications (SSD) which can produce different kind of adaptations compared to WGD [14]. The increasing number of sequenced genomes, extending the taxons sampling, can help providing new insights into the evolutionary history and the origin of genes (i.e. from SSD or WGD) and the encoded protein function [15].

In this context, the present study first aimed at revisiting and clarifying the evolutionary history of g6pc encoding genes among vertebrates, taking into consideration all genomes sequenced and indexed in Ensembl Genome Browser (www.ensembl.org). Since comparative studies of gene expression are critical for establishing links between divergent gene expression and divergence of a particular phenotype, we thus proposed to examine the evolution of the spatial *g6pc* expression pattern in vertebrates using the powerful PhyloFish database [16]. Indeed, this database provides a transcriptomic repertoire of 10 tissues and organs in 23 different ray-finned fish species including two holosteans (i.e. an infraclass of Neopterygii which diverged before the Ts3R) and 21 teleosts among which 6 salmonids. By supplementing these data by in vivo analysis of *g6pc* in a jawless fish (lamprey) and by the in silico expression pattern of several Sarcopterygii *g6pc* (xenopus, chicken, rat, mouse, sheep, cow, babouin olive, rhesus macaque and human) provided by the gene-annotation portal BioGPS [17] and by the Expression Atlas [18], we intend to provide new insights into the expression domains of *g6pc* in vertebrates. The latter results should provide new trails of investigations (i.e. new territories of expression) to better understand the atypical regulation of duplicated *g6pc* genes by dietary carbohydrates in salmonid [6, 19].

## Results

### Phylogenetic and synteny analysis of g6pc evolutionary history among vertebrates

By analysing vertebrate genomes available in Ensembl (Release 86, October 2016), we found one gene related to the glucose-6-phosphatase encoding gene (*g6pc*) in lamprey, a jawless vertebrate; 2 in spotted gar, a holostei; between 1 and 2 in amphibians, reptiles and owes and one in mammals, and 2 to 3 in teleosts (except for the cod) (Additional file 1: Figure S1). Based on the automatic annotation provided in Ensembl, *g6pc* appeared to be lost in duck, chicken, platypus, opossum, Tasmania devil, armadillo, pig, tree shrew and orangutan. Using blast and syntenic (Genomicus v85.01) approaches we found one *g6pc* gene in each of these species (Additional file 1: Figure S1). Moreover, we identified two genomic sequences, scaffold 606 and contig 60457, bearing two *g6pc* related genes in the elephant shark (*Callorhinchus milii*) genome (http://esharkgenome.imcb.a-star.edu.sg/blast/), a cartilaginous fish (Chondrichthyes). The size of the scaffold/contig bearing these sequences was not sufficient to cover the whole gene length. The length of the deduced amino acid sequence was therefore not sufficient for phylogenetic analysis (see below). However, these truncated elephant shark sequences were submitted to a BLAST search against the spotted gar genome in the Ensembl database. The hit with the highest score matched the spotted gar *g6pca* when blasting the scaffold 606 sequence (Evalue: 1.7 e^-117^, score: 419) and spotted gar *g6pcb* gene when blasting the contig 60457 sequence (Evalue: 1.7 e^-62^, score: 234) (data not shown).

**Fig. 1:**
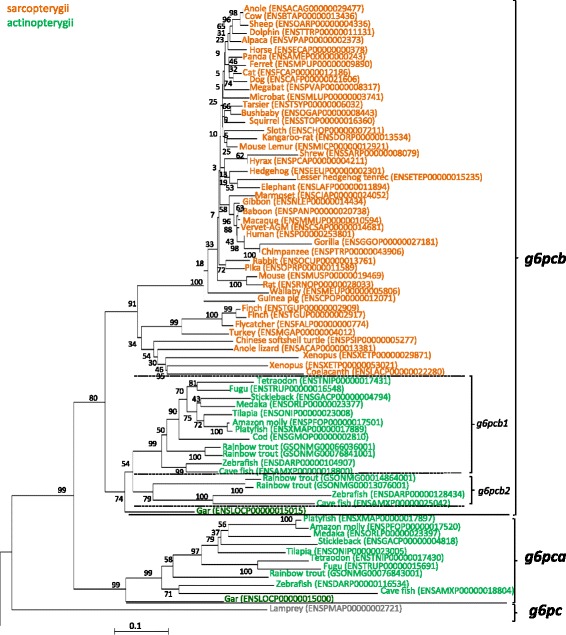
Phylogenetic analysis of glucose-6-phosphatase encoding genes in vertebrates. Phylogenetic analyses were performed using MEGA package version 6 software [48], as previously described [49]. The phylogenetic tree was built by the neighbor-joining (NJ) method. The reliability of the inferred tree was estimated by the bootstrap method with 1000 replications. Mammalian and teleosts g6pc3 protein sequences were used to root the tree. All accession numbers (from Ensembl, or Genoscope databases) are specified in parentheses. Sarcopterygii species are written in *orange* and Actinopterygii species in *green*

We then selected representative Ensembl species for each class of vertebrate as well as sequences previously found in the rainbow trout genome [6], a salmonid fish, and performed a phylogenetic analysis. This analysis showed that the *g6pc* gene identified in lamprey stemmed from the base of the phylogenetic tree (Fig. 1). For the spotted gar, one *g6pc* gene (ENSLOCP00000015000) rooted with teleosts *g6pca* genes while the other (ENSLOCP00000015015) was orthologous to both *g6pcb1* and *g6pcb2* genes previously described in teleosts [6], defining them as *g6pca* and *g6pcb*, respectively.

This phylogenetic tree also revealed that all *g6pc* identified in Sarcopterygii (including the two genes found in xenopus, anole and zebra finch) and in the coelacanth grouped together and were orthologous to *g6pcb* related genes in holostei and teleosts.

**Fig. 2:**
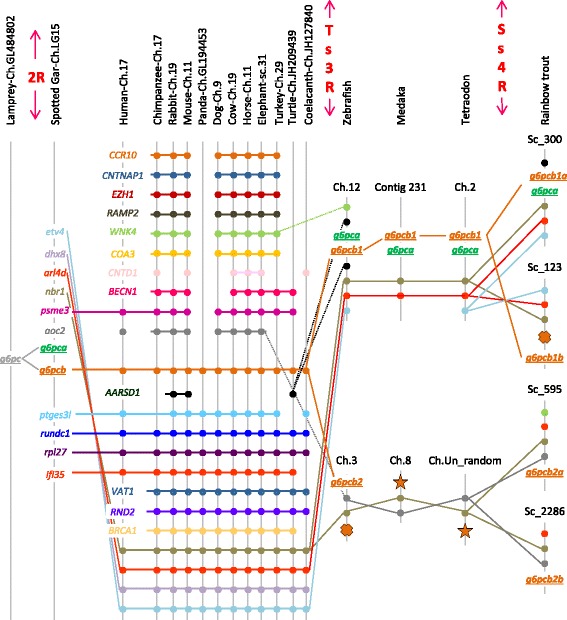
Conserved synteny around the *g6pc* loci in vertebrates. Data were collected with Genomicus software version 86.01, and we annotated *g6pc* genes according to our phylogenetic analysis following ZFIN nomenclature guidelines. The teleosts synteny was modified from Marandel et al. (2015). Remnants of *g6pca* and *g6pcb2* were identified by red crosses and stars, respectively. Ch., chromosome; sc., scaffold, cont., contig; 2R, Second whole genome duplication; Ts3R, teleost-specific whole genome duplication; Ss4R, salmonid-specific whole genome duplication

We then performed a synteny analysis to clarify the evolutionary history of *g6pc* in vertebrates. The chromosome bearing *g6pc* in lamprey was too short to provide relevant synteny information. However, our analysis, in addition to the one previously proposed by Marandel et al. (2015) in teleosts, showed that *g6pc* genes found in Sarcopterygii were all included in a syntenic group conserved across vertebrates (Fig. 2). To identify potential remnants of the *g6pca* gene in Sarcopterygii, the spotted gar *g6pca* cDNA sequence was submitted to a Blat search (http://genome.ucsc.edu/index.html) against sequenced genomes of Sarcopterygii. Unfortunately, we were unable to find any significant hit in analysed genomes. Thus, in order to confirm the identity of the only *g6pc* gene conserved in Sarcopterygii and to strengthen our phylogenetic analysis, we performed a protein percentage identity matrix and an alignment. The identity matrix (Additional file 5: Table S2) and associated alignment (Additional file 2: Figure S2) confirmed that g6pc proteins in Sarcopterygii shared higher sequence identity with the spotted gar *g6pcb* than with its *g6pca* paralog. Remarkably g6pc protein, whatever the orthologous gene by which it is encoded (i.e., *g6pca*, *g6pcb1* or *g6pcb2* gene) or the species considered, shared high sequence conservation around the binding (red arrows, Additional file 2: Figure S2) and the active (yellow arrows, Additional file 2: Figure S2) sites of the enzyme (sites described in Uniprot, http://www.uniprot.org). It is also noteworthy that the exon/intron structure of *g6pc* genes was conserved among Osteichthyes evolution as all *g6pc* genomic structures displayed 5 exons and 4 introns (the lamprey *g6pc* only displayed 4 exons and 3 introns) (data not shown).

A Blat analysis was also conducted in teleosts, in order to identify remnants of genes suspected to have been lost in different orders (such as the duplicate of *g6pca* in zebrafish and trout and also *g6pcb2* in euteleostei) [6]. We identified one hit (red cross, Fig. 2) on the zebrafish chromosome 3 (at the location 32,780,748-32,780,748; 49 amino acids hit sharing 72% identity with zebrafish g6pca) and one on the rainbow trout scaffold_123 (at the location 1,457,501–1,457,677; 58 amino acids hit sharing 66% identity with zebrafish g6pca) for *g6pca* remnant but none on the scaffold_595 or the scaffold_2286 of trout. In addition, one hit (red stars, Fig. 2) on the medaka chromosome 8 (at the location 19,097,860–19,097,879; 19 nucleotide bases hit sharing 100% identity with the trout *g6pcb2b*) and one on the tetraodon chromosome Un_random (at the location 79,811,101–79,811,120; 20 nucleotide bases hit sharing 100% identity with the trout *g6pcb2b*) were found for *g6pcb2* remnant.

### Evolution of g6pc expression domains in vertebrates

**Fig. 3:**
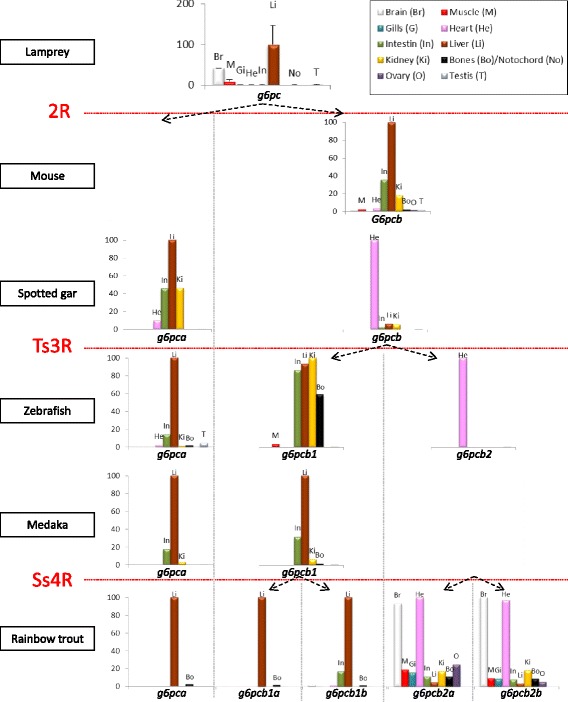
Tissue expression profiles of *g6pc* genes in vertebrates. Excepted for lamprey, relative expression of *g6pc* genes was calculated as the percentage of the maximum rpkm (number of reads per kilobase per million reads) value per species. These data were obtained from BioGPS database for the mouse and from Phylofish database for other species. Relative abundance of *g6pc* mRNA in lamprey was established by RT-qPCR analysis and expressed as means ± SE (*n* = 10)

The PhyloFish database [16], a unique resource providing comprehensive expressed gene repertoires in 23 ray-finned fish species, was used to explore the evolution of the spatial expression of *g6pc* genes. In order to have an overall view across the vertebrate subphylum, we also supplemented this analysis by RT-qPCR in lamprey and by *in silico* data analysis for several Sarcopterygii (xenopus, chicken, rat, mouse, sheep, cow, babouin olive, rhesus macaque and human) provided by the gene-annotation portal BioGPS [17] and by the Expression Atlas [18]. The expression domains of *g6pc* genes of representative species are presented in Fig. 3 and data obtained from PhyloFish in the 23 ray-finned fish and in other Sarcopterygii are displayed in Additional file 3: Figures S3 and Additional file 4: Figure S4, respectively.

Our results showed that *g6pc* was preferentially expressed in the brain and in the liver of lamprey (Fig. 3). Based on *g6pca* expression patterns in PhyloFish species, we observed that this gene was mainly expressed in the liver of holostei (i.e., spotted gar and bowfin) and teleosts. In addition, *g6pca* was also highly expressed in intestine and kidney of the spotted gar, and to a lower extend in zebrafish and medaka. As for *g6pcb* genes, we found in the BioGPS and the Expression Atlas databases that *G6pcb* in Sarcopterygii (xenopus, chicken, rat, mouse, sheep, cow, babouin olive, rhesus macaque and human) was also preferentially expressed in liver, intestine and kidney whereas in holostei, in addition to these latter three organs, it was mainly expressed in heart. *g6pcb2* was also showed to be preferentially expressed in heart in cypriniformes (zebrafish), characiformes (Cave Mexican Tetra and Surface Mexican Tetra) and esociformes (Northern pike) as well as in salmonid (Rainbow trout, Brown trout and Brook trout). In salmonid, *g6pcb2* duplicated genes were also highly expressed in brain and in a lesser extent in all other analysed organs. In contrast, but similarly to *g6pca*, *g6pcb1* was preferentially expressed in liver, intestine and kidney in all teleost species excepted for salmonids in which *g6pcb1* duplicated genes were mainly expressed in the liver.

### Expression pattern of g6pc genes in brain and heart of rainbow trout fed a high carbohydrate diet

**Fig. 4:**
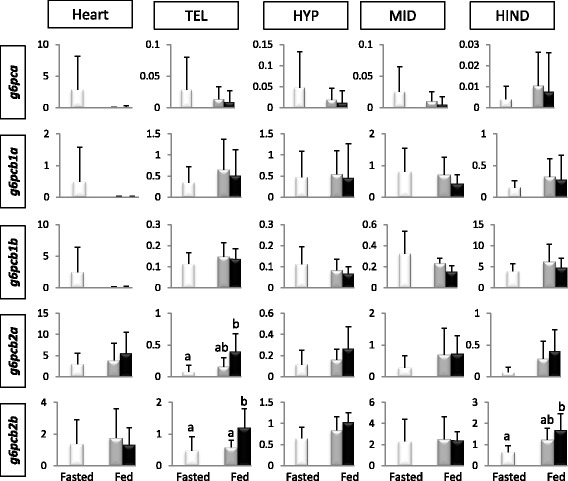
Relative expression of *g6pc* genes in brain regions and heart of rainbow trout. Relative abundance of *g6pc* paralogs in fasted trout (*white* bars), trout fed with the NoCHO (*gray* bars) or the HighCHO (*black* bars) diet. Data are expressed as means ± SE (*n* = 6). Different letters over bars indicate significant differences between conditions (*P* < 0.01). TEL, telencephalon; HYP, hypothalamus; MID, midbrain; HIND, hindbrain

As we found that *g6pcb2* genes were preferentially expressed in brain and heart in salmonids, we investigated the expression pattern of *g6pc* genes in heart and different areas of brain of rainbow trout fed diets without (NoCHO) or with a high amount (HighCHO) of carbohydrates. This analysis revealed that *g6pcb2a* and *g6pcb2b* mRNA levels were higher in the telencephalon of trout fed the HighCHO diet compared to fasted trout (Fig. 4). The same expression pattern was also monitored in the hindbrain for *g6pcb2b*. Moreover, *g6pcb2b* mRNA level was higher in the telencephalon of trout fed the HighCHO diet compared to trout fed the NoCHO diet (Fig. 4).

## Discussion

**Fig. 5:**
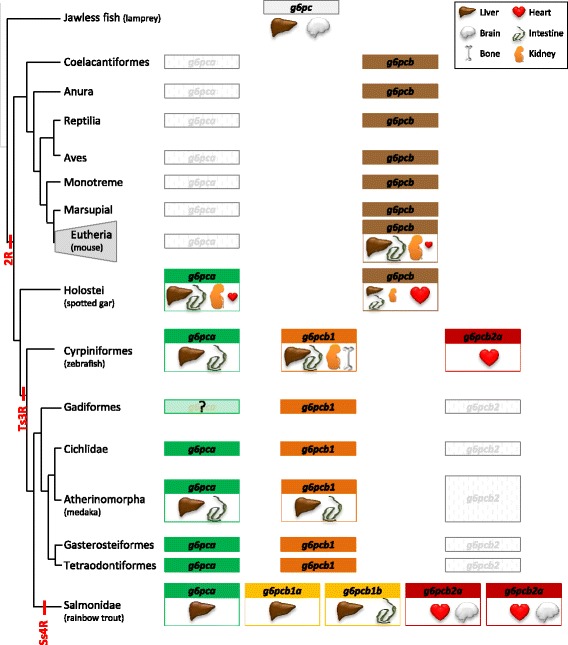
Summary of the evolutionary history and spatial expression patterns of *g6pc* genes in vertebrates

g6pc duplications happened by successive rounds of WGD during vertebrate evolution. All the results related to the evolution of *g6pc* in vertebrates and obtained in this study are summarised in Fig. 5.

The first critical step in elucidating the evolutionary history of *g6pc* gene was to accurately identify orthologous genes in investigated species. Our phylogenetic and synteny analyses demonstrated that there was only one *g6pc* gene in lamprey, a jawless fish, but also only one gene in Sarcopterygii whereas at least two orthologous *g6pc* genes were found in Chondrichthyes and Actinopterygii. While it is commonly accepted that 1/2R occurred before the divergence of Chondrichthyes [20], these results thus strongly suggested that *g6pc* duplicated before or around Osteichthyes/Chondrichthyes radiation, giving rise to *g6pca* and *g6pcb* probably as a consequence of the 2R. Our phylogenetic analysis led to the conclusion that *g6pca* was lost after this duplication in Sarcopterygii. The broad view of *g6pc* evolution including all vertebrates presented here improved the understanding of the timing of *g6pc* duplication. Indeed, in a previous study [6] focused on teleosts, we only considered Sarcopterygii species as outgroup to elucidate *g6pc* evolution in this infraclass. We thus concluded that, due to the presence of only one *g6pca* gene and two *g6pcb* genes in the genome of several teleosts, a duplication of *g6pc* occurred before or around teleost radiation followed by an additional duplication of *g6pcb* that occurred at least in the genome of the common ancestor of the euteleosts, salmonids, and ostariophys. By the identification of *g6pca* remnants in zebrafish and rainbow trout and *g6pcb2* remnants in medaka and tetraodon, our present results also showed that *g6pca* and *g6pcb* duplicated as a consequence of the Ts3R. These results also showed that one *g6pca* duplicate was lost in teleost genomes and confirmed the findings previously proposed by Marandel et al. (2015) that *g6pcb2* was lost in Percomorpha.

In conclusion, together with the finding previously reported in teleosts [6], our results strongly suggest that *g6pc* diversity originates, at least in part, from successive whole genome duplications from 2R to Ss4R. In addition, duplicated *g6pcb* genes found in Xenopus (tropicalis), finch and anole lizard probably arose from a lineage-specific duplication.

### g6pc functionally diverged among vertebrates through sub- and neo-functionalisation of their spatial pattern of expression

The emergence of new genes through duplication is now considered as a driver process of evolution. Indeed, these new genes are genetic material available for supporting the rise of new molecular and cellular functions, and can play an important role in phenotypic variability [7–9]. Duplicated genes fixed in a genome can conserve the function of the ancestral gene or functionally diverge [10]. Collected information about ancestral state is critical for the classification of this divergence into sub- and neofunctionalisation [21]. We thus analyzed the spatial expression pattern of *g6pc* genes in vertebrates completing information obtained from the PhyloFish, the BioGPS and the Expression Atlas databases by RT-qPCR analysis in lamprey (summary available in Fig. 5).

Our results indicate that, following 2R, the *g6pc* expression in the brain found in lamprey was lost in tetrapods as well as in holostei whereas the hepatic expression of *g6pca* and *g6pcb* remained. By contrast, intestine, kidney, and heart were identified as new territories of *g6pca* and *g6pcb* expression in both tetrapods and holostei. However, a g6pc activity was previously described in kidney (a tissue not analyzed here) [22] as well as in brain of mammals [23]. The missing result in databases concerning *g6pc* expression in the brain of at least mammals (to our knowledge *g6pca* and *g6pcb* expression in brain has never been studied in holostei) was probably due to the faint expression of this gene in this tissue [23]. For other tissues, our results in lamprey (especially for the brain expression) and tetrapods were in accordance with published works [5, 22, 24]. Together with previous published data, our results suggest that after the 2R, *g6pc* duplicates underwent neofunctionalization in heart and intestine.

After the Ts3R, our results showed that *g6pca* remained mainly expressed in intestine, liver and kidney as well as *g6pcb1*. In contrast, *g6pcb2* was preferentially found expressed in the heart in species even though the expression in intestine, liver and kidney was anyway detected. To our knowledge, *g6pcb2* expression pattern has never been studied in non-salmonid teleosts, thus our results strongly suggested for the first time that *g6pcb1* and *g6pcb2* were subfunctionalised after Ts3R. Moreover, data collected from the PhyloFish database were in favor of the loss of the expression of *g6pc* genes in brain of non-salmonid teleosts after the Ts3RTGD. As the functional role of *g6pc* in brain and heart has never been elucidated, further functional studies are required to investigate the possible functional schemes of such a loss as well as of the loss of *g6pcb2* in percomorpha species.

Finally, in salmonids *g6pca* was almost exclusively expressed in the liver as well as *g6pcb1a* whereas *g6pcb1b* mRNA was found mainly in intestine in addition to liver. By contrast, *g6pcb2a* and *g6pcb2b* displayed a ubiquitous expression but were mostly found in brain and heart whereas they were faintly expressed in liver. As *g6pcb2* was not found in the brain of non-salmonid teleosts, this finding was in favor of a neofunctionalisation of *g6pcb2* genes in the brain of salmonids.

However, we cannot rule out the possibility that the new expression of *g6pc* gene in brain was gained independently in lamprey and salmonid lineages.

In conclusion, this spatial expression pattern analysis showed that *g6pc* diverged during vertebrate evolution mainly through sub- or neo-functionalization at lest in terms of expression domains. Our results also showed that important protein domains (i.e. binding and catalytic domains) promoting the activity of the enzyme were conserved during vertebrate evolution as well as the exon/intron structure of *g6pc* genes. Together, our findings suggest that *g6pc* orthologs/paralogs diversified at the level of their regulation as previously supposed by epigenetic investigations carried out in salmonids [25].

In addition, the most surprising result of our study was the report of *g6pc* mRNA expression in heart and brain. Indeed, these two organs need glucose as fuel energy to sustain their metabolic requirements, they are thus considered as glycolytic rather than gluconeogenic organs. Even if *g6pc* activity was previously detected in heart and brain of mammals [23, 24, 26–29] and brain of lamprey [30–33], its role remains poorly understood. An unexpected result was that salmonid *g6pcb2* paralogs were found mostly expressed in brain and heart and poorly found in liver. Indeed these genes were previously shown to display an atypical up-regulation of their mRNA levels in the liver of trout fed a high carbohydrate diet [6, 19]. In order to better understand the role of these genes we thus investigated their expression patterns in different brain areas and in heart in trout fed with or without dietary carbohydrates.

### g6pcb2b gene was up-regulated by dietary carbohydrates the in telencephalon of trout

The discovery of new g6pc encoding genes, i.e. *g6pcb2* genes, in salmonids [6] and the present results, showing their neofunctionalisation and preferential expression in brain and heart, suggested new function(s) of these genes in these tissues. We thus investigated their expression patterns in different areas of brain and in heart of fasted trout and of trout fed a high or a no carbohydrate diet.

Our results showed that there were no differences in mRNA levels of *g6pc* paralogs in heart whatever the nutritional status of trout. In brain regions, we observed that *g6pca* and duplicated *g6pcb1* remained stable whereas *g6pcb2b* increased in telencephalon of rainbow trout fed the carbohydrate-enriched diet. This result suggested the functionalisation of *g6pcb2b* genes in this brain area related to the content of carbohydrates in the diet. What functions may cover this change in enzyme expression? There is evidence for possible relationships of g6pc/endogenous glucose production pathway with glucose homeostasis.

In rainbow trout brain the presence of glucosensing mechanisms was demonstrated in hypothalamus and hindbrain [34–38] and they relate to the control of food intake as well as to counter-regulatory responses to changes in circulating levels of glucose (see review by Soengas [39]). These mechanisms result in enhanced glycolytic potential in response to increased levels of extracellular glucose. Since hindbrain and probably telencephalon are glucosensing areas in this species [36, 37, 40] we would expect *a priori* a decrease in the mRNA levels of *g6pc* genes in response to increased levels of carbohydrates in the diet. However, this response did not occur in the present experiment thus not supporting that the responses observed relate to glucosensing capacity. This is further supported by the fact that hypothalamus (the main glucosensing area in brain of vertebrates including rainbow trout) did not show any response to changes in dietary carbohydrate content. Therefore, the changes observed in response to carbohydrate intake may relate to functions different than those elicited by canonical glucosensors on food intake regulation and counter-regulatory mechanisms (Soengas 2014).

Several studies reported the presence of active g6pc enzyme in brain of vertebrates including agnathes [30, 31], elasmobranchs [41], tetrapods [26–28], and teleosts [42]. In lampreys, available studies suggest that this tissue could be, in a certain extent, autonomous from glucose supplied through the blood as demonstrated by endogenous glucose production either from gluconeogenesis or glycogenolysis, a process in which glucose-6-phosphatase action is involved [26, 28, 30, 31] thus suggesting a possible function of *g6pc*. In teleosts, levels of glycogen in brain are considerably lower than in lamprey even though the enzymes involved in gluconeogenesis and glycogen metabolism are also present and functional as previously demonstrated in rainbow trout [37, 38, 43]. The role of gluconeogenesis in teleost brain is however still an enigma though it could relate to glucose production under conditions of limited glucose supply such as under food deprivation. In fact, studies in food-deprived rainbow trout [2] showed increased glycogenolytic capacity and thus presumably g6pc activity in the whole brain. Changes observed in the present study in response to increased dietary carbohydrate levels are not consistent with this hypothesis since decreased rather than increased expression of *g6pc* is expected. These findings suggest that *g6pcb2* may endorse another function in the brain and be involved in specific unknown process related to specific functions of the telencephalic (olfaction, social behavior, stress response…) area.

Finally, the present *g6pcb2b* expression pattern in telencephalon was previously observed in the liver of trout subjected to the same experimental plan [6]. The up-regulation of this gene in trout fed a high carbohydrate diet mimicked expression profile of *gckb* in the telencephalon (data not shown), one of the two ohnologous genes that encodes for the first enzyme of the glycolysis pathway (the glucokinase), as well as what was observed in the liver. Based on this, it was hypothesised that a futile glucose/glucose-6-phosphate cycling, involving g6pc and gck, may take place in the liver in order for example to protect cells from oxidative stress [6]. The existence of such a futile cycle in the brain remains under debate in mammals [44–46] but has never been studied in teleosts to date.

In conclusion, *g6pcb2b* was atypically up-regulated by dietary carbohydrates in a localized brain area. Its role however remained questioning and further investigations such as knock-out of *g6pcb2* genes are needed to better understand the function(s) they acquired after duplication.

## Conclusions

In conclusion, the present study proposes for the first time a thorough analysis of the evolutionary history of glucose-6-phosphatase genes in vertebrates. Our results showed that the diversity of *g6pc* genes results from successive rounds of whole genome duplication that occurred in vertebrates (excepted for species-specific *g6pcb* duplication in tetrapods) followed by the loss of some duplicates in specific lineages. We also report that the spatial expression pattern of duplicated genes significantly diverged in the different vertebrate lineages investigated. Finally, this evolution of spatial expression patterns of *g6pc* genes in vertebrates demonstrated that *g6pcb2* genes, which were shown to be atypically regulated by dietary carbohydrates in salmonid liver, were mainly expressed in brain and heart. We showed that *g6pcb2b* was up-regulated by dietary carbohydrates in telencephalon of rainbow trout and not in other brain areas. The increased capacity for endogenous glucose production suggested by this change in expression is not compatible with glucosensing and may relate to specific unknown processes specific of these brain areas. Together, our results strongly suggest that the fixation and the divergence of *g6pc* duplicated genes during vertebrate evolution may lead to adaptive novelty and probably to the emergence of particular phenotypes related to glucose homeostasis. As previously proposed for the glycolytic pathway [47], gluconeogenesis should have thus subsequently evolved by gene duplication and divergence of *g6pc*.

## Methods

### In silico analysis

Orthologous g*6pc* genes and related protein sequences were identified in the Genomicus software program, version 01.01 (http://www.genomicus.biologie.ens.fr/genomicus-trout-01.01/cgi-bin/search.pl) and collected from Ensembl (Ensembl release 86 - October 2016, http://www.ensembl.org). *G6pc* genes in duck, chicken, platypus, opossum, Tasmania devil, armadillo, pig, tree shrew and orangutan were found using the BLAST tool in Ensembl (Additional file 1: Figure S1). Elephant shark (*Callorhinchus milii*) *g6pc* genes were identified by blasting the *g6pc* spotted gar sequences in the Elephant shark genome project (http://esharkgenome.imcb.a-star.edu.sg) database.

The phylogenetic analyse (Fig. 1) was performed using the MEGA package version 6 software [48], as previously described [49]. The phylogenetic tree, based on full-length amino acid deduced sequences, were built using the Neighbor-Joining (NJ) method and confirmed by the Minimum evolution method (data not shown). The reliability of the inferred trees was estimated using the bootstrap method with 1000 replications. G6pc3 protein sequences from mammals and teleosts were used to root the tree.

The Genomicus software program, version 01.01 (http://www.genomicus.biologie.ens.fr/genomicus-trout-01.01/cgi-bin/search.pl) was used to confirm their identities by establishing synteny analysis (Fig. 2). The presence of *g6pca* and *g6pcb2* gene remnants was queried against the zebrafish, medaka and the tetraodon genomes with BLAT [50] tool proposed by the UCSC Genome Browser (https://genome.ucsc.edu) and against the rainbow trout genome using the BLAT tool proposed by the Genoscope (http://www.genoscope.cns.fr/trout/).

Protein alignment (Additional file 2: Figure S2) and the Percentage Identity Matrix established among G6pc sequences (Additional file 5: Table S2) were performed using MUSCLE software (www.ebi.ac.uk/Tools/msa/muscle).

Exon/intron structure was obtained from the Ensembl or the Genoscope databases or identified by alignment of the predicted mRNA of rainbow trout *g6pc* genes and the genomic sequence of zebrafish *g6pc* (data not shown).

Finally, tissue expression profiles of *g6pc* genes (Fig. 3 and Additional file 3: Figure S3) were identified in the PhyloFish database using the Blast tool [16]. In the case of ohnologs which possess highly similar sequences (such as *g6pcb1a* and *g6pcb1b* in trout; [6]), we considered fragments that included SNPs specific to each gene in PhyloFish database in order to distinguish the expression of each of them.

Expression data in several Sarcopterygii (xenopus, chicken, rat, mouse, sheep, cow, babouin olive, rhesus macaque and human) were also collected from the BioGPS annotation portal (http://biogps.org) and the Expression Atlas (http://www.ebi.ac.uk/gxa/home).

### Total RNA extraction and cDNA synthesis

Brain, muscle, liver, gills, heart, intestine, notochord, testis and ovary from ten lampreys and different part of brain from 18 trout were sampled and frozen in liquid nitrogen and then kept at −80 °C (more details are given in “fish and experimental design” section).

Relative *g6pc* genes mRNA levels in lamprey and trout tissues was determined by quantitative real-time RT-PCR. Samples were homogenized using Precellys®24 (BertinTechnologies, Montigny-le-Bretonneux, France) as previously described [6, 19] and total RNA was extracted in Trizol reagent (Invitrogen) according to the manufacturer’s instructions. Luciferase control RNA (Promega),10 pg per 1.9 mg of tissue, was added to each sample to allow for data normalization as previously described [19]. Total RNA (1 μg) was used for cDNA synthesis. The Super-Script III RNAse H-Reverse transcriptase kit (Invitrogen) was used with random primers (Promega, Charbonniéres, France) to synthesize cDNA. For real-time RT-PCR assays, the Roche Lightcycler 480 system was used (RocheDiagnostics, Neuilly-sur-Seine, France). The protocol conditions for real-time RT-PCR have been published previously [6]. The primer sequences used in real-time RT-PCR assays for luciferase and *g6pc* genes in trout analysis were previously described [6]. The forward and reverse primers for g6pc lamprey analysis were 5’- TCGTCTACTTCCCCATCTGC-3’ and 5’- AGGTGATGGGGAACTGCTG-3’ respectively. For trout samples, data were subsequently normalized to the exogenous luciferase transcript abundance in samples diluted at 1:76 using the E method (Light Cycler software). For lamprey samples, data were subsequently normalized to the geometric mean of β-actin (forward : 5’- GCCAACCGTGAAAAGATGACA-3’, reverse: 5’- GGATGGCGACGTACATTGC-3’), 5S (forward : 5’-CCTACGACCATATCACCCTGA -3’, reverse: 5’-TTCCCAGGTAGTCTCCCATC-3’) and exogenous luciferase transcript abundance in samples diluted at 1:76 using the E method (Light Cycler software) as previously proposed by Vandesompele et al. (2002) [51].

### Fish, experimental design and analytical methods

#### Rainbow trout

Juvenile rainbow trout (~70 g body mass) were obtained from a local fish farm (A Estrada, Spain), randomly distributed into six 100 litres tanks (15 fish per tank) and maintained for 1 month under laboratory conditions and 12 L:12D photoperiod (lights on at 08:00 h, Lights off at 20:00 h) in dechlorinated tap water at 15 °C. Fish were fed once daily (09.00 h) to satiety with commercial dry fish pellets (Dibaq-Diproteg SA, Spain; proximate food analysis was 48% crude protein, 14% carbohydrates, 25% crude fat, and 11.5% ash; 20.2 MJ/kg of feed). The experiments described comply with the Guidelines of the European Union Council (2010/63/UE), and of the Spanish Government (RD 55/2013) for the use of animals in research, and were approved by the Ethics Committee of the Universidade de Vigo.

Following acclimation fish were deprived of food for 4 days. On the next day two fish per tank were captured, anesthesised with MS-222 buffered to pH 7.4 with 1 M sodium bicarbonate, and sacrificed by decapitation. After sacrifice brain was dissected to obtain regions (hypothalamus, hindbrain, midbrain, and telencephalon) that together with heart were snap-frozen in liquid nitrogen and stored at -80 °C. The remaining fish in tanks were then fed with either the NoCHO or the HighCHO diet (triplicate tanks per diet, Additional file 6: Table S1) twice a day at 2.5% live weight for four days, then they were anesthesised and sampled as described above 6 h after the last meal (*n* = 9 fish per diet, 3 per tank). Gut content of the sampled animals was systematically checked to confirm that the fish sampled had consumed the diet.

#### Lamprey

Investigations were conducted according to the guiding principles for the use and care of laboratory animals and in compliance with French and European regulations on animal welfare (Décret 2001-464, 29 May 2001 and Directive 2010/63/EU, respectively). Ten lampreys were obtained from a local wholesale sea-fish merchant (groupe Aguirre barrena, Saint Vincent de Tyrosse, France). Fish were anesthetised and killed immediately as described above after been bought and brain, muscle, liver, gills, heart, intestine, notochord, testis and ovary were sampled and frozen in liquid nitrogen and then kept at −80 °C.

#### Analytical methods

The chemical composition of the diets was analysed using the following procedures: 1) dry matter was determined after drying at 105 °C for 24 h, 2) protein content (N × 6.25) was determined by the Kjeldahl method after acid digestion, 3) fat was determined by petroleum ether extraction (Soxtherm), 4) gross energy was determined in an adiabatic bomb calorimeter (IKA, Heitersheim Gribheimer, Germany), 5) ash content was determined by incinerating the samples in a muffle furnace at 600 °C for 6 h, and 6) starch content was measured by an enzymatic method (InVivo Labs, France).

### Statistical analysis

Normality of distributions was assessed using the Shapiro-Wilk test. Data were then analyzed by a Kruskal-Wallis non-parametric test following by a Tukey test as a post-hoc analysis. Data were analysed using the R software (v.3.3.1)/R Commander package.

## Additional files

Additional file 1: Figure S1. *g6pc* genes number across vertebrates. Phylogenetic analysis modified from Ensembl species tree (http://www.ensembl.org/info/about/species_tree.pdf). In green, g6pc genes automatically annotated in Ensembl; in blue, new identification of *g6pc* genes and location. (PDF 83 kb)

Additional file 2: Figure S2. Alignment of vertebrates g6pc protein sequence. Red and yellow arrows indicate predicted binding and active sites respectively. Relative expression was calculated as the percentage of the maximum rpkm (number of reads per kilobase per million reads) value per species. NF, not found; Br, brain, M, red muscle; Gi, gills; He, heart; Int, intestine; Li, liver; Kid., kidney; Bo, bones; Ov, ovary; T, testis; E, embryo. (PDF 692 kb)

Additional file 3: Figure S3. Relative expression of *g6pc* genes in all species available in PhyloFish database (PDF 467 kb)

Additional file 4: Figure S4. Relative expression of *g6pc* gene in several Sarcopterygii species from BioGPS and Expression Atlas databases. When data were from Expression Atlas, name(s) of experiment(s) used were mentioned as written in the database. NA : not applicable (no data available) (PDF 380 kb)

Additional file 5: Table S2. Percentage Identity Matrix between tetrapods and lamprey/spotted gar g6pc proteins. (DOCX 17 kb)

Additional file 6: Table S1. Formulation and proximate composition of the two experimental diets used (NoCHO and HighCHO diets) in this experiment. (DOCX 19 kb)

## Abbreviations

G6pc: Glucose-6-phosphatase
Ss4R: Salmonid-specific whole genome duplication
SSD: Small-scale duplications
Ts3R: Teleost-specific whole genome duplication
