# Supplementary figures and images for: Evolutionary history of glucose-6-phosphatase encoding genes in vertebrate lineages: towards a better understanding of the functions of multiple duplicates
Source: BMC Genomics. 2017 May 2;18:342. doi: 10.1186/s12864-017-3727-1 (PMC5414149; doi:10.1186/s12864-017-3727-1)

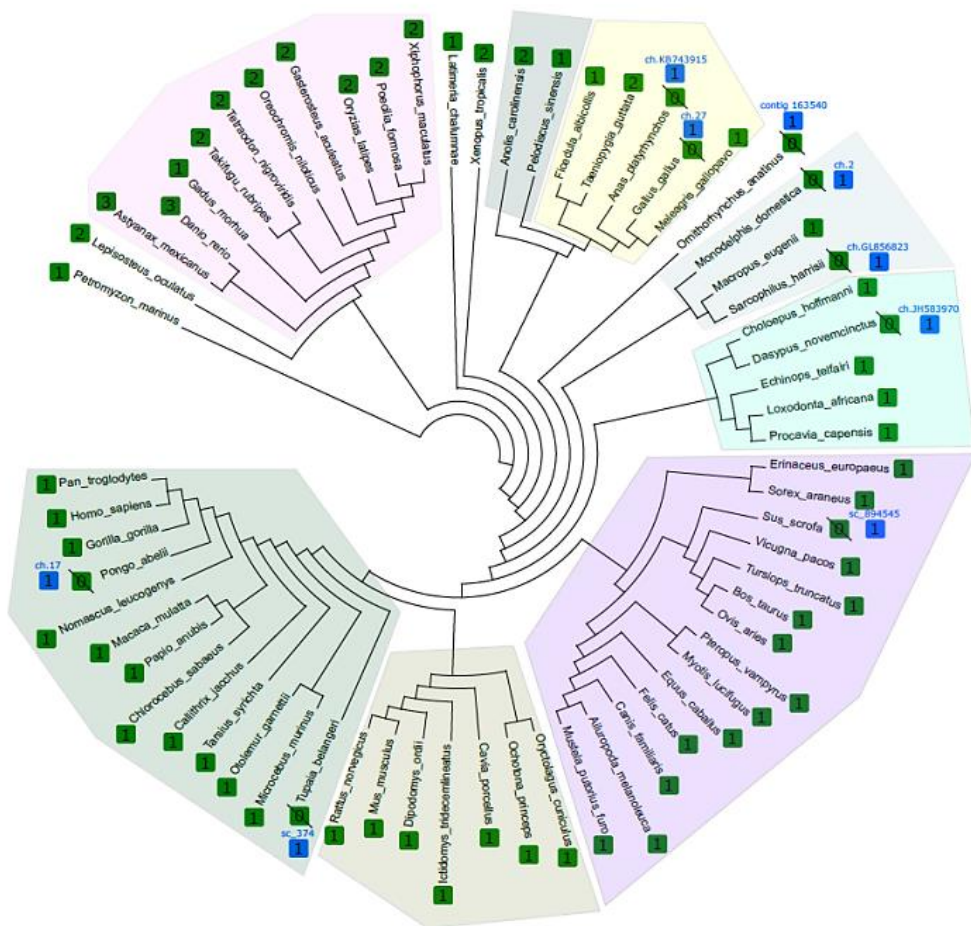

Supplement: Supplementary file 1 — g6pc genes number across vertebrates. Phylogenetic analysis modified from Ensembl species tree (http://www.ensembl.org/info/about/species_tree.pdf). In green, g6pc genes automatically annotated in Ensembl; in blue, new identification of g6pc genes and location. (PDF 83 kb) [file 12864_2017_3727_MOESM1_ESM.pdf]
