# Supplementary material for: Evolutionary history of glucose-6-phosphatase encoding genes in vertebrate lineages: towards a better understanding of the functions of multiple duplicates
Source: BMC Genomics. 2017 May 2;18:342. doi: 10.1186/s12864-017-3727-1 (PMC5414149; doi:10.1186/s12864-017-3727-1)
Supplement: Supplementary file 2 — Alignment of vertebrates g6pc protein sequence. Red and yellow arrows indicate predicted binding and active sites respectively. Relative expression was calculated as the percentage of the maximum rpkm (number of reads per kilobase per million reads) value per species. NF, not found; Br, brain, M, red muscle; Gi, gills; He, heart; Int, intestine; Li, liver; Kid., kidney; Bo, bones; Ov, ovary; T, testis; E, embryo. (PDF 692 kb) [file 12864_2017_3727_MOESM2_ESM.pdf]

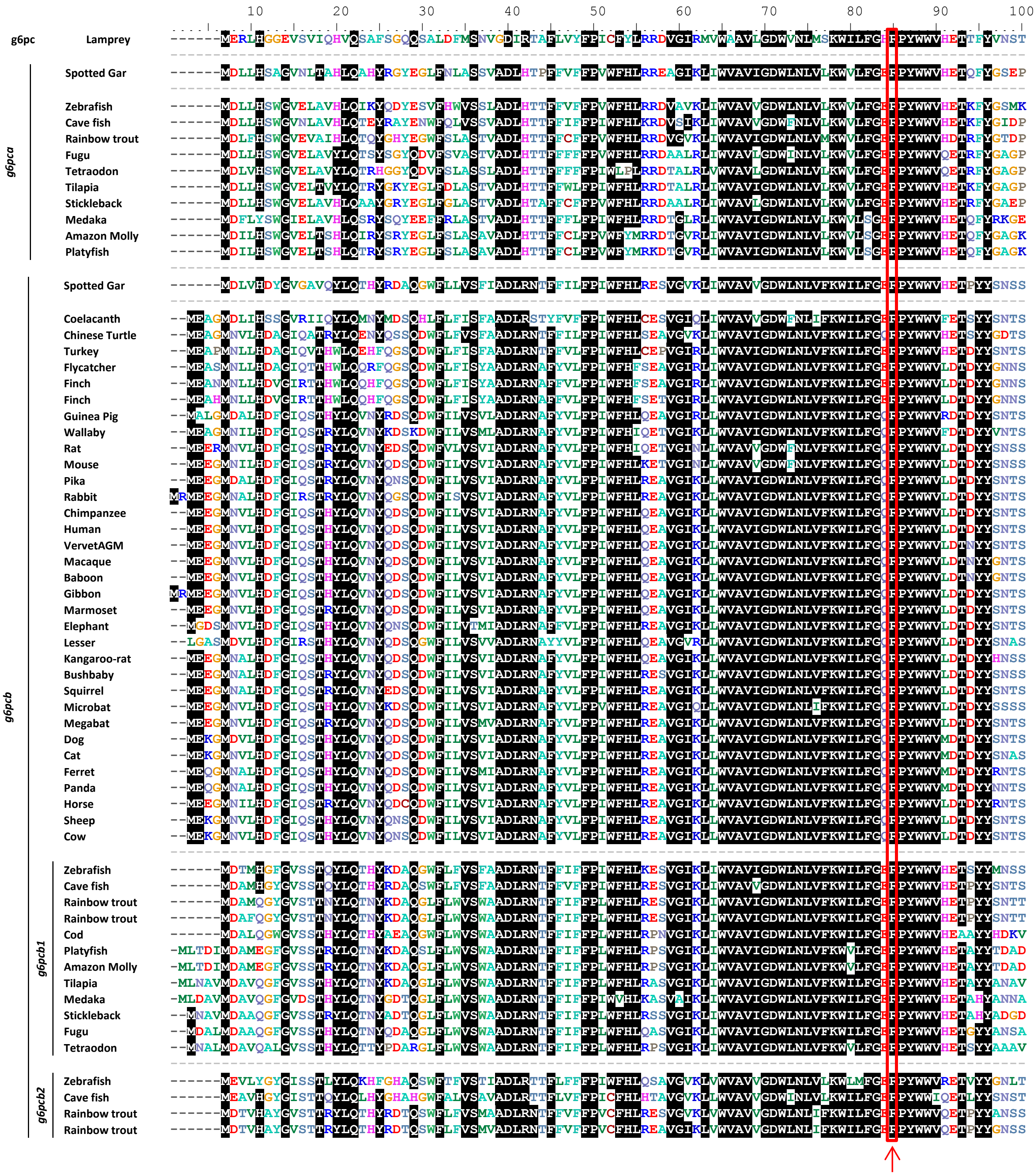

10 20 30 40 50 60 70 80 90 100
g6pc
Lamprey
g6pca
Spotted Gar
Zebrafish
Cave fish
Rainbow trout
Fugu
Tetraodon
Tilapia
Stickleback
Medaka
Amazon Molly
Platyfish
g6pcb
Spotted Gar
Coelacanth
Chinese Turtle
Turkey
Flycatcher
Finch
Finch
Guinea Pig
Wallaby
Rat
Mouse
Pika
Rabbit
Chimpanzee
Human
VervetAGM
Macaque
Baboon
Gibbon
Marmoset
Elephant
Lesser
Kangaroo-rat
Bushbaby
Squirrel
Microbat
Megabat
Dog
Cat
Ferret
Panda
Horse
Sheep
Cow
g6pcb1
Zebrafish
Cave fish
Rainbow trout
Rainbow trout
Cod
Platyfish
Amazon Molly
Tilapia
Medaka
Stickleback
Fugu
Tetraodon
g6pcb2
Zebrafish
Cave fish
Rainbow trout
Rainbow trout

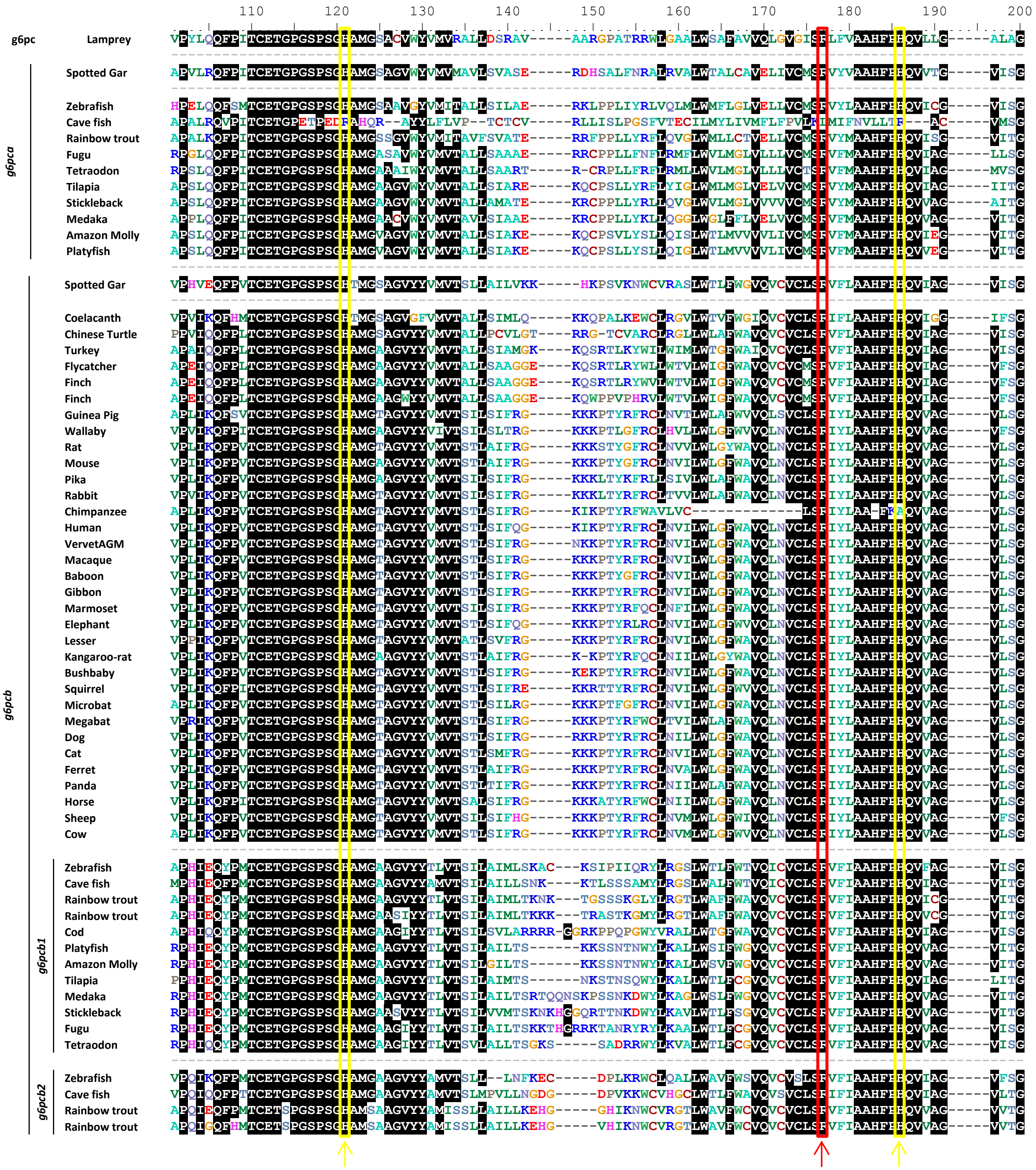
110 120 130 140 150 160 170 180 190 200
g6pc
Lamprey
g6pca
Spotted Gar
Zebrafish
Cave fish
Rainbow trout
Fugu
Tetraodon
Tilapia
Stickleback
Medaka
Amazon Molly
Platyfish
g6pcb
Spotted Gar
Coelacanth
Chinese Turtle
Turkey
Flycatcher
Finch
Finch
Guinea Pig
Wallaby
Rat
Mouse
Pika
Rabbit
Chimpanzee
Human
VervetAGM
Macaque
Baboon
Gibbon
Marmoset
Elephant
Lesser
Kangaroo-rat
Bushbaby
Squirrel
Microbat
Megabat
Dog
Cat
Ferret
Panda
Horse
Sheep
Cow
g6pcb1
Zebrafish
Cave fish
Rainbow trout
Rainbow trout
Cod
Platyfish
Amazon Molly
Tilapia
Medaka
Stickleback
Fugu
Tetraodon
g6pcb2
Zebrafish
Cave fish
Rainbow trout
Rainbow trout

```
                              210       220       230       240       250       260       270       280       290       300
g6pc    Lamprey          MVVADLFGSVSCIHNAPLSRYVGTSAAIFALALGFYLALLAVGVDLLWTVAFAKKWCLHPEWVHLDSTPFAGLVRNLGVLAGTGLALNSGLHRDALAGAR

        Spotted Gar      MIVAEAFSRVQWIYSASLKRYLLTTLFLLVFALGFYLLLRVLGVDLLWTLEKAKRWCVRAEWVHMDTTPFASLLRNLGTLFGLGLGLHSPLSSGSHSGKR

g6pca   Zebrafish        IIVAETFSRVQWIYSASLKKYFSITLFLLSFAVGFYVLLKALGVDLLWTLEKAQKWCINPAWVLMDATPFASLLRNMGTLFGLGLGLHLSHYDPGS-SKK
        Cave fish        IIVAEVFSRQKWIYRASLQKYFSITVFLLVFAVGFYVLLKVLGVDLLWTLEKAQKWCNNPSWVNMDTSPFASLLRNMGTLFGLGLGLHWPLCS--E-SKL
        Rainbow trout    IMVAEAFSRVQWIYGASLKKYFYTTLFLLSFAVGFYELLKAIGVDLLWSLEKAQKWCVRAEWVYMDSTPFASLLRNMGTLFGLGLGLHSPLY---T-ENK
        Fugu             VLVAEVVSRQKWIYGASLRNYACTTLFLTSFAIGFYLLLKAVGVDLLWTMEKAQRWCVRPEWVHLDTTPFASLLRNMGTLLGLGLGLQLGLGLGLQ-LPQ
        Tetraodon        VLVAEVVSRQKWIYGASLRSYICTTLLLTSFAVGFYLLLRAVGVDLLWTMEKAQKWCARPEWVHLDTTPFASLLRNMGSLLGLGLGLHSPPSRSSS-KPG
        Tilapia          TLVAEVVSKEKWIYSASLKKYFLITLFLTSFAVGFYVLLKALDVDLLWTMEKAQKWCIRPEWVHLDSAPFASLLRNMGSLFGLGLGLHSPFYKTT--KMK
        Stickleback      VLVAEAVSKEKWILSAGMKKYFHTTLFLTAFAVGFYLVLRSVGVELLWTLDKAQKWCARPEWVHLDSTPFASLLRNMGTLMGLGLGLHSPLHAQN--EKK
        Medaka           VLVAKIVSKSKWMYNASMNKYFFITFHLTFFAVGFYLLLRTLGVDLLWTLDKAQKWCVKPEWIHLDSTPFASLLRNMGSLLGLGMGLHSPIHNGV--KKK
        Amazon Molly     IVVAGMVSKKKWIYSASMSKYLLITLFLTFFAIGFYLLLKIVSVDLLWTLDKAQKWCIKPEWIHLDTTPFASLLRNMGSLFGLGLGLHSPFYHGI--KKN
        Platyfish        IVVAGMVSKRKWMYSASMSKYLLITLFLTFFAIGFYLLLKIVGVDLLWTLDKAQKWCLKPEWIHLDTTPFASLLRNMGSLFGLGLGLHSPFYHGV--KKN

        Spotted Gar      MIVAEAFSRVQWIYSASLKRYLLTTLFLLVFALGFYLLLRVLGVDLLWTLEKAKRWCVRAEWVHMDTTPFASLLRNLGTLFGLGLGLHSPLYSESCSGKR

g6pcb   Coelacanth       MVIAKIITHTRFIHKATLKRYLYIILFLFFFNLGSYLLLKALGVDLLWSIEKAKRWCARADWIHMNSTPFASLIRNLGVLFGLGLALNSQIYTEICKGKQ
        Chinese Turtle   MVVAEAFEHIHAIYNASLRRYLGTTLFLFGFALGFYLLLKALGVDLLWTLEKAERWCDRPEWIHIETTPFAGLLRNLGILFGLGLALNSQMYLESCKGKR
        Turkey           MAVAETFQHIHSIYHASLHRYLGITFFLFSFALGFYLLLRVLGVELLWTLEKAQKWCAHPEWVHIDTTPFASLLRNLGILFGLGLALSSHMYLESCRGKQ
        Flycatcher       MAVAKTFQHIRFIYRASFHRYLGITLFLFSFTLGFYLLLWTFGVDLLWTLEKAQKWCSRPEWVHIDTTPFASLLRNLGILFGLGLALNSHMYQESSRLKQ
        Finch            MAVAKTFQHVRCIYQASFHRYLGITIFLFSFTLGFYLLLWTFGVDLLWTLEKAQKWCSNPEWVHIDTTPFASLLRNLGVLFGLGLALNSHLYQESSRLKQ
        Finch            MAVAKTFQHVRCIYQASFHRYLGITIFLFSFTLGFYLLLWTFGVDLLWTLEKAQKWCSNPEWVHIDTTPFASLLRNLGVLFGLGLALNSHLYQESSRLKQ
        Guinea Pig       IAVAETSYHVRGIYNASLRRYILVTGFLFGFAVGFYLLLRVLGVDLLWTLEKARRWCERPEWVHIDTTPFASLLKNLGTLLGLGLALHSGMYRQSCGSRL
        Wallaby          IAVAETFGHIQSIYNASLKKYIVITFFLFSFAIGFYLMLKAMGIDLLWTLEKAKRWCEQPEWVHIDTTPFASLLKNLGTLFGLGLALNSNMYKQSCKGKQ
        Rat              IAVAETFSHIRGIYNASLQRYCLITFFLFGFALGFYLLLKGLGVDLLWTLEKAKRWCERPEWVHLDTTPFASLFKNLGTLLGLGLALNSSMYRKSCKGEL
        Mouse            IAVAETFSHIRGIYNASLRKYCLITIFLFGFALGFYLLLKGLGVDLLWTLEKAKRWCERPEWVHLDTTPFASLFKNLGTLLGLGLALNSSMYRKSCKGEL
        Pika             IAVAETFCHIQSIYNASLKKYFLITFFLFSFAIGFYLLLKGLGVDLLWTLEKAKRWCERPEWVHIDTTPFASLLKNLGTLFGLGLALNSRMYRESCKGKL
        Rabbit           IAVAEAFHHIRGIYNASLQKYFLITFFLFSFAIGFYLLLKGLGVDLLWTLEKAKRWCERPEWVHIDTTPFASLLRNLGTLFGLGLALHSSMYRESRKGRL
        Chimpanzee       IAVAETFSHIHSIYNASLKKYFLITFFLFSFAIGFYLLLKGLGVDLLWTLEKAQRWCEQPEWVHIDTTPFASLLKNLGTLFGLGLALNSSMYRESCKGKL
        Human            IAVAETFSHIHSIYNASLKKYFLITFFLFSFAIGFYLLLKGLGVDLLWTLEKAQRWCEQPEWVHIDTTPFASLLKNLGTLFGLGLALNSSMYRESCKGKL
        VervetAGM        IAVAETFSHIHSIYNASLKKYFLITFFLFSFAIGFYLLLKGLGVDLLWTLEKAQRWCERPEWVHIDTTPFASLLKNLGTLFGLGLALNSSMYRESCKGKL
        Macaque          IAVAETFSHIHSIYNASLKKYFLITFFLFSFAIGFYLLLKGLGVDLLWTLEKAQRWCERPEWVHIDTTPFASLLKNLGTLFGLGLALNSSMYRESCKGKL
        Baboon           IAVAETFSHIHSIYNASLKKYFLITFFLFSFAIGFYLLLKGLGVDLLWTLEKAQRWCERPEWVHIDTTPFASLLKNLGTLFGLGLALNSSMYRETCKGKL
        Gibbon           IAVAETFSHIHSIYNASLKKYFLITFFLFSFAIGFYLLLKGLGVDLLWTLEKAQRWCERPEWVHIDTTPFASLLKNVGTLFGLGLALNSSMYRESCKGKL
        Marmoset         IAVAETFSHIHGIYNASLKRYFLVTFFLLSFAVGFYLLLKGLGVDLLWTMEKAQRWCERPEWIHIDTTPFASLLKNMGTLFGLGLALNSRMYRESCKSKL
        Elephant         IAVAETFHHIRSIYNASLKKYSFITFFLFSFAIGFYLLLKGLDVDLLWTLEKAKRWCERPEWVLIDSTPFASLFRNLGTLFGLGLALNSSMYRESCKGKL
        Lesser           IAVAEAFCRTQRIYNTSLKKYSLVTCFLFSVAIGFYLVLKGLDVDLLWTLEKAKRWCERPEWVLIDSTPFASLFRNLGTLFGLGLALNSAMYRASCRGQL
        Kangaroo-rat     IAVAETFNHIQSIYKASLKKYFFITLFLFSFAIGFYLLLKGLGVDLLWTVEKAKRWCQQPEWVHIDTTPFASLLKNLGTLFGLGLALNSSMYKESCKGKS
        Bushbaby         IAVAETFHHIRSIYNASLKKYFLITFFLFSFAIGFYLVLKGLGVDLLWTLEKAKRWCERPEWVHIDTTPFASLLKNLGTLFGLGLALNSSMYRESCRGSL
        Squirrel         IAVAETFRHIQSIYNASLKKYVLITFFLFSFAIGFYLLLKGLGVDLLWTLEKAKRWCERPEWVHIDTTPFASLLKNLGTLFGLGLALNSNMYRESCKGKL
        Microbat         IAVAETFRHIQGIYHASLKKYFLITFFLFSFAIGFYLLLKGLGVDLLWTVEKAKRWCERPEWVHIDTTPFASLLKNLGTLFGLGLALNSSMYRESCKGQL
        Megabat          IAVAETFRNIQSIYNASLKKYFLITFFLFSFAIGFYLLLKGLGIDLLWTLEKAKRWCERPEWVHIDTTPFASLLKNVGTLFGLGLALNSSMYRESCKGKL
        Dog              IAVAETFRHIQSIYNASLKKYFLITFFLFSFAIGFYLLLKGLGVDLLWTLEKARRWCERPEWVHIDTTPFASLLKNVGTLFGLGVALNSSMYRESCKGKL
        Cat              IAVAETFRHIQSIYNASLKKYFFITFFLLSFAIGFYLLLKGLGVDLLWTLEKARRWCERPEWVHIDTTPFASLLKNVGTLFGLGLALNSSMYRESCKGTL
        Ferret           IAVAETFRHIQSIYNASLKKYFLVTFFLFGFAIGFYLLLKGLGVDLLWTLEKAKRWCQRPEWVHIDTTPFASLLKNVGTLFGLGLALNSSMYRESCKGKL
        Panda            IAVAETFRHTQSIYNASLKKYFLITFFLFSFAIGFYLLLKGLGVDLLWTLEKAKRWCERPEWVHIDTTPFASLLKNLGTLFGLGLALNSSMYRESCKGRL
        Horse            IAVAETFCRIQSIYNASLKKYFLITFFLFSFAIGFYLLLKGLGVDLLWTLEKAKRWCEQPEWVHIDTTPFASLFKNLGTLFGLGLALNSSMYRESCKGKL
        Sheep            IAVAETFRHIQSIYNASLKKYFLITCFLFSFAIGFYLLLKWLGVDLLWTLEKAKRSCERPEWVHIDTTPFASLLKNLGTLFGLGLALNSSMYRESCKGKL
        Cow              IAVAETFRHIQSIYNASLKKYFLITCFLFSFAIGFYLLLKWLGVDLLWTLEKAKRRCERPEWVHIDTTPFASLLKNLGTLFGLGLALNSSMYRESCKGKL

g6pcb1  Zebrafish        MIVAEAFNRQKWIYSASLKNYFNVTLFLLSFAVGLYVLLKALGVDLLWTLEKAQKWCANPAWVHLDTTPFASLLRNMGTLFGLGLGLHSPLYTES---KK
        Cave fish        MIVAEVFNRQKWIYQASLQKYFNITLFLLSFAVGLYVLLKAMGVDLLWTLEKAQKWCVNPAWVHLDTTPFASLLRNMGTLFGLGLGLHSPLYTES---KR
        Rainbow trout    MVVAEAFNRTQWIYGASLKKYFYTTLFLLSTAVGFYVLLKALGVDLLWTLEKAQKWCVRAEWVHMDSTPFASLLRNMGTLFGLGLGLHSPLYTES---KN
        Rainbow trout    IVVAEAFNRTQWIYGASLKKYFYTTVFLLSFAVGFYVLLKALGVDLLWTLEKAQKWCVRAEWVHMDSTPFASLLRNMGTLFGLGLGVHSPLYTES---KK
        Cod              MIVAEIFDRVQWIYSASMRSYLLTTLFLTSFAVGFYLLLRALGVDLLWTLEKAQRWCVKAEWVHMDSTPFASLLRNMGTLFGLGLALHSPLYTES---KR
        Platyfish        MIVAEAFNRTQWIYSASMKKYFYTTLFLTSFAVGFYVLLKAVGVDLLWTLEKAQKWCVRPEWVNLDTTPFASLLRNMGTLFGLGLGLHSPLYTET---KK
        Amazon Molly     MIVAEAFNRTQWIYSASMKKYFYTTLFLTSFAVGFYVLLKAVGVDLLWTLEKAQKWCVRPEWVHLDTTPFASLLRNMGTLFGLGLGLHSPLYTET---KK
        Tilapia          MIVAEAFNRTQWIYSASMKKYFYTTLFLTSFAVGFYVLLKALGVDLLWTMEKAQKWCIRPEWVHLDTTPFASLLRNMGTLFGLGLGLHSPLYTET---KK
        Medaka           MIVAEAFNRTQWIYSASMKKYFYTTLFLTSFAVGFYLLLKSLGVDLLWTLDKAQKWCARPEWVHLDTTPFASLLRNMGTLFGLGLGLHSPLYSET---KR
        Stickleback      IIVAEAFNRTQWIYSASMKKYFYTTLFLTSFAVGFYLVLRSVGVDLLWTLEKAQKWCVRPEWVNLDSTPFASLLRNMGTLFGLGLGLHSPLYAEA---KR
        Fugu             VIVAEAFNRTRWIYGASMRKYFCTTLFLTSFAVGFYLLLKAVGVDLLWTMEKAQRWCVRPEWVHLDTTPFASLLRNMGTLFGLGLGLHSPLYAET---KK
        Tetraodon        MLVAEAFNRTRWIYGASLRSYVCTTLLLTSFAVGFYLLLRAVGVDLLWTMEKAQRWCARPEWVHLDTTPFASLLRNMGTLFGLGLGLHSPLYTQS---KQ

g6pcb2  Zebrafish        IAVAKAFNRVSWIYSASLKGYMHSILCLVFVALGLYAVLDAASIDPYWSLMKAQKWCIQSEWVHLDTTPFAGLLRNTGALFGLGLSLHLPTHGDL---QK
        Cave fish        IAVAKVVNRASWIYSATLRSYIHTTLFLLSFAIGLYTLLEVASIDPHWSLSKAQRWCLHPDWVRLDTTPFAGLLRNTGALFGLGLSLHLSMMIET---ET
        Rainbow trout    ILVAEIFDRIQWIYRASLRCYVYTTLSLLSFAVGFYLVLRGLGVDLLWTLDKAQRWCQRPQWVHIDTTPFASLLRNTGTLLGLGLGLHSPLYAEA---RR
        Rainbow trout    QWRAPTFDRVQWIYRASLRRYVYTTLSLLSFAVGFYLVLRGLGVNLLWTLDKAQRWCQWPQWVHIDTTPFASLLRNTGTLLGLGLGLHSPLYAEA---RR
```

```
                                  310        320        330        340        350        360        370        380
g6pc    Lamprey          GEA----PAFRAVCAVAAVVTLRLYDGVKVPSDDELAFYVLTFCKSLLVPLSALALLPYTLRHLLDAGTRHHKLA------

g6pca   Spotted Gar      GHS----APFRLGCITASLLLLQLLDLVHFSSERELLFYLLSFCKSAAVPLITVALAPCVISRLCPRQGKRQK--------

        Zebrafish        HTC----ICFRIGCIGVSLVLLQLLDKMTFSSSSQ-LFYLLSFCKSAVALLLPTALVPGIMYWITQKSKHEKDM-------
        Cave fish        SSS----AAFRISCIGVSLVLLQLLDSLSFSSDNQATFYLLSFSKSAAALLVPTTLVPGGISLFLLGKKDEKTS-------
        Rainbow trout    NSS----IPFRVGCITVSLLLQILDGLTFSSRDQAMFYVLSFSKSAAALFIPTALVPGGLSWIFPGSGAAKLKF------
        Fugu             SGS----VSSKAGRITASLLLQLLDGWTFSSEDLLTFYFLSFAKSAVALLIPTLLVPWALCCVFERNARSKDL-------
        Tetraodon        S-------ASRTGRIAASLLLQLWDMWTFSSENLLIFYFLSFAKSMVALLIPTRLVPWALCWSPERSSSTKDL-------
        Tilapia          SMN----VSFRIGCIVISVSLHLLDGWTFSPENHMTFYALSFGKSAIALLIPTTLVPWALSKIYPVKTEGKNL-------
        Stickleback      ETS----SGFKMGCIIVSLFLLQLLDGWTFSHENLMTFYLLSFGKSAVALFIPTALVPCVLHWIC---KENKVL-------
        Medaka           SST----TAFKIGSITISLFLLQLLDEWTFSSENHLIFYILSFCKSAIALLIPTAVVPWALCRIFTGKTDGKNL-------
        Amazon Molly     -AN----AGFKMGCIMSALFLLHLLDGWTFSSDNHLTFYILSFGKSAFALLIPTTLIPWALCRICKGMELDKNL-------
        Platyfish        -AN----AGFKMGCIMTSLFLLHLLDGWTFSSDNHTTFYILSFGKSAFALLIPTTLIPWALCRICKGLELDKNL-------

g6pcb   Spotted Gar      GH----SAPFRLGCIAVSLSLLHLFDSFKPPTHMVALFYLLSFCKSAAVPLATVGIIPYCLSGALS-AHSKKTL--------

        Coelacanth       GKQ----LSFRLSCIVSSLVALHLFDSFKPPTKVELLFYILSFCKSAAVPLATIGIIPYCVFHILSKQQKKAL--------
        Chinese Turtle   GQR----LPFRLSCIAASLLVLHLFDSFDLPTDRELLFYLLSFCKNAAAHLCAVALIPYCVAQVLRRGHEKMR--------
        Turkey           GHQ----LSFRLGCAAASLLVLHLFDAFRLPSHMQLLFYVLSFCKSAAVPLATVGLIPYCISQFLATQDKKGV--------
        Flycatcher       GQQL---LPFRLGCIAASLLTLHIFDAFKPPSHTQLLFYALSFCKSAAVPLAAVGLIPYCLSQLLGTQDKKAA--------
        Finch            GQQ----LPFRLGCVAASLLILHVFDAFKPPSHIQLLFYALSFCKSAAVPLATVGLIPYCLSQLLATQHKKGG--------
        Finch            GQQ----LPFRLGCVAASLLILHVFDAFKPPSHVQLLFYALSFCKSAAVPLAAVGLIPYCLSQLLATQHKKAA--------
        Guinea Pig       HG-----LPFRLGCIGASLVLLHLLDTWKPPAQVELVFYILSFCKSAAVPLASVSLIPYCLAQLLGRPHGKVL--------
        Wallaby          KKQ----LPFRLCCIVASLVILHLFDAFKPPSQVEMLFYALSFCKSAAVPLTSVSLIPYCFSQILGQSQKKFL--------
        Rat              RKS----LPFRLACIVASLGLLHLFDSLKPPSQIESIFYILSFCKSATVPFASVSLIPYCLARLLGQTHKKSL--------
        Mouse            SKL----LPFRFACIVASLVLLHLFDSLKPPSQVELIFYILSFCKSATVPFASVSLIPYCLARILGQTHKKSL--------
        Pika             SKR----LPFRLSCIVTSLVLLHLFDSLKPPSQVELIFYALSFCKSAVVPLASVSLIPYCLARALGQTHKKSL--------
        Rabbit           NRR----LPFRLGCIVVSLVLLHLLDSLKPPSHVELVFYALSFCKSAAVPLASVSLVPYCLARVLGQTHKKSL--------
        Chimpanzee       SKW----LPFRLSSIVASLVLLHVFDSLKPPSQVELVFYVLSFCKSAVVPLASVSVIPYCLAQVLGQPHKKSL--------
        Human            SKW----LPFRLSSIVASLVLLHVFDSLKPPSQVELVFYVLSFCKSAVVPLASVSVIPYCLAQVLGQPHKKSL--------
        VervetAGM        GKW----LPFRLSSIVASLVLLHLFDSLKPPSQVELVFYVLSFCKSAVVPLASVSVIPYCLAQVLGQPHKKSL--------
        Macaque          GKW----LPFRLSSIVASLVLLHLFDSLKPPSQVELVFYVLSFCKSAVVPLASVSVIPYCLAQILGQPHKKSL--------
        Baboon           GKW----LPFRLSSIVASLVLLHLFDSLKPPSQVELVFYVLSFCKSAVVPLASVSVIPYCLAQVLGQPHKKSL--------
        Gibbon           SKW----LPFRLSSIVASLILLHLFDTLKPPSQVELVFYVLSFCKSAVVPLASVSIIPYCLAQVLGQPHKKSL--------
        Marmoset         SKW----LPFRLSCIVASLVLLHLFDSLKPPAQVELVFYILSFCKSATVPLASVSVIPYCLAQVLGQPHKKSL--------
        Elephant         RMW----LPFRLSCILASLIFLHLFDSLKPPSQIELLFYVLSFCKSAAVPLASASLIPYCLMWVLGQPDKKSL--------
        Lesser           GRW----LPFRLGCILASLVFLHFFDSLRPPSQIELLFYGLSFCKSAAVPLMAASLVPYCLSRVLGQPGKKSL--------
        Kangaroo-rat     SKA----LSFRLSCIVLSLVLLHLFDSLKPPSKIELIFYALSFCKSATVPLASASLIPYCLSRFLGQSHKKSL--------
        Bushbaby         SKW----LPFRLSCIVTSLILLHLFDSLKPPSQIELIFYVLSFCKSMTVPLASVSLIPYCITRILGQSHKKSL--------
        Squirrel         SKW----LPFRLSCIVASLVLLHLFDSLKPPSQIELIFYILSFCKSATVPLASVSVIPYCLARVLGQSHKKTL--------
        Microbat         SKR----VPFRLGCIVASLILLHLFDSLKPPSQVEMIFYVLSFCKSAAVPLASVSLIPWCLARILGQSDKKSL--------
        Megabat          SKW----YPFRLSCIVTSLVLLHLFDSLKPPSQIELIFYILSFCKSAVVPLASVSLIPYCLSRILSQPDKKFL--------
        Dog              SKW----FPFRLSCIVVSLILLHLFDSLKPPSQTELIFYTLSFCKSAAVPLASVSLIPYCLARVFDQPDKKSL--------
        Cat              SKW----FPFRLSCIVVSLILLHLFDSLKPPSQIELIFYVLSFCKSAAVPLASVSLIPYCLARVLGQPDKKSL--------
        Ferret           SKW----FPFRLCCIVVSLVLLHLFDSLKPPSQVELIFYVLSFCKSAAVPLASVSLIPYCLARVLGQPDKKSL--------
        Panda            GKW----SLFRVSCIVVSLILLHLFDSLKPPSQVELIFYALSFCKSAAVPLASVSLIPYCLARVLGQPDKKSL--------
        Horse            SKC----FPFRLSCILASLVLLHLFDSLKPPSQTELIFYVLSFCKSAAVPLASVSLIPYCLAWVLGQPDKKSL--------
        Sheep            SKS----FPFRLSCIVASLVLLHLFDSLKPPSQIELIFYVLSFCKSAAVPLASVSLIPYCLARVLGQPNKKTL--------
        Cow              SKW----FPFRLSCIVASLVLLHLFDSLKPPSQIELIFYVLSFCKSAAVPLASVSLIPYCLAWVLGQPNKKTV--------

g6pcb1  Zebrafish        SS----NAFVRITCIIASLLLLHLFDSIKPPTHTAALFYLLSFCKSATVPLVTVSIIPYCLSGALG-LQSKKQL-------
        Cave fish        SG----SAPFRIGCIVSTLFLLHLFDSIKPPTHTAALFYLLSFCKSATVPLVTVSIIPYCLSGAMS-LQSKKQL-------
        Rainbow trout    ISG---TTLFRTGCIVTSLLLLHLFDSFKPPTHTAAVFYLLSFCKSATVPLATVSIIPYCVSGALSSVQSKKYL-------
        Rainbow trout    NS----STPFRVGCIVTSLLLLHLFDSFKPPTHTAALFYLLSFCKSATVPLATVSIIPYCMSGALSSIQSKKHL-------
        Cod              SS----GAVAKAGCIVSSLLLLHLFDSFKPPTHTAALYYLLSFCKSATVPLVAGSIVPYCVNSALS-LQGKKGL-------
        Platyfish        SS----SASARAGCILSSLFLLHLFDSFKPPTHTAALFYLLSFCKSATVPLVTVSIIPYCVNRALS-QHSKKGM-------
        Amazon Molly     SS----SASARAGCILSSLFLLHLFDSFKPPTHTAALFYLLSFCKSATVPLVTVSIIPYCVNRALS-QHSKKGM-------
        Tilapia          SS----SPLVKAGCIISSLLLLHLFDSFKPPTHTAALFYLLSFCKSATVPLVTVSIIPYCVNRSLN-QHNKKAV-------
        Medaka           SN----SATVKAGCIISSLVLLHLFDSFKPPTHTAALFYLLSFCKSATVPLVTVSIIPYCVSRALT-SQGKKFM-------
        Stickleback      SA----GRSSKVGCVVASLVLLHLFDSFKPPTHTAALFYLLSFCKSATVPLVVVSIVPYCVNGALG-LQSKKGAWPRDYED
        Fugu             SN----SKLAKVVCVVTSLVLLHLFDSFKPPTHTAALFYLLSFSKSATVPLVTISIIPYCVSGVLN-LHRKAAL-------
        Tetraodon        SS----SGRARAACAVASLVLLHLFDSVKPPTHTAALFYLLSFCKSATVPLVTISIIPYCVSAALS-LHRKAA--------

g6pcb2  Zebrafish        NKNCGNSAFYRLTCTAATLPLLSLLDSFRPPTSTHALFYTLSFCKSATVPLTTISFVPYCISTLANAYRRMKAH------
        Cave fish        CS-YRDSTIYRLFCALATLLLLSLLDSFRPPDHTQALFYALSFCKSATVPLTTVGLVPYCATAVLNMYQRRKCT------
        Rainbow trout    VG---GGATYRLACVGATLLLLHLLDSFRPPAHTRALFYLLSFCKSATVPLATVGIVPYCVAGAAGQNGKKH-------
        Rainbow trout    VG---GGATYRLACVGATLLLLHLLDSFRPPAHTRVLFYLLSFCKSATVPLATVGIVPYCVAEAAGRNGKKHX--------
```
