# Supplementary material for: Evolutionary history of glucose-6-phosphatase encoding genes in vertebrate lineages: towards a better understanding of the functions of multiple duplicates
Source: BMC Genomics. 2017 May 2;18:342. doi: 10.1186/s12864-017-3727-1 (PMC5414149; doi:10.1186/s12864-017-3727-1)
Supplement: Supplementary file 4 — Relative expression of g6pc gene in several Sarcopterygii species from BioGPS and Expression Atlas databases. When data were from Expression Atlas, name(s) of experiment(s) used were mentioned as written in the database. NA : not applicable (no data available) (PDF 380 kb) [file 12864_2017_3727_MOESM4_ESM.pdf]

Species	Experiment	Brain	Intestin	Kidney		Liver	Heart	Muscle	Lung	Ovary	Testis	Bone
**Xenopus tropicalis** (ENSXETP00000029871)	Barbosa-Morais et al	0	NA	5		100	2	0	NA	NA	NA	NA
**Chicken**	Merkin et al	0	0,16	89		100	1	0	0,5	NA	0	NA
	Barbosa-Morais et al	0	NA	100		91,2	1	0,08	NA	NA	NA	NA
**Mouse**	Mammalian Kaessmann	0	NA	100		17,9	0	NA	NA	NA	0	NA
	Vertebrates	0	NA	73,25		100	0,44	0	NA	NA	NA	NA
	Soumillon et al	0	NA	NA		100	NA	NA	NA	NA	0	NA
	BioGPS	7,8	3	86		100	0,06	0,07	2	0,06	0,06	NA
**Rat**	Yu - female, adult	4	NA	100		76	3	NA	7	NA	NA	NA
	Yu - male, adult	4	NA	100		87	3	NA	6	NA	6	NA
	BioGPS	1,28	1	100		NA	1,5	1,5	NA	NA	NA	1,59
**Sheep**				cortex	kidney							
	Roslin - male, adult	NA	1	100	NA	59	NA	NA	0	NA	0	NA
	Roslin - female, juvenile	NA	NA	100	NA	NA	NA	0	0	0	NA	NA
	Roslin - female, adult	NA	NA	NA	NA	100	NA	NA	1	0	NA	NA
	Jiang et al - Texel	0	NA	NA	100	48	0	NA	0,16	0,08	NA	NA
**Cow**	Liao	NA	0,24	93		100	NA	NA	1	NA	NA	NA
**Babouin olive**	Pipes	0	0	39		100	0	0	0	NA	NA	NA
**Rhesus macaque**	Merkin et al	0	NA	79		100	0	0	0	NA	0	NA
	Kaessmann lab	0	0	30		100	0	NA	NA	NA	0	NA
**Human**	FANTOM5 project - adult	9,6	NA	100		NA	1	NA	0	0	0	0
	Uhlen's Lab	NA	19	39		100	0	0	0	0	0	0
	Illumina Body Map	0	NA	9		100	0	0	0	0	0	NA
	ENCODE (M. Snyder lab)	0	NA	5		100	0	NA	0	0	0	NA
	Mammalian Kaessmann	NA	NA	8		100	0	NA	NA	NA	0	NA
	BioGPS	1,4	35,1	18		100	3	2	2	1	1	NA
