# Supplementary material for: Evolutionary history of glucose-6-phosphatase encoding genes in vertebrate lineages: towards a better understanding of the functions of multiple duplicates
Source: BMC Genomics. 2017 May 2;18:342. doi: 10.1186/s12864-017-3727-1 (PMC5414149; doi:10.1186/s12864-017-3727-1)
Supplement: Supplementary file 5 — Percentage Identity Matrix between tetrapods and lamprey/spotted gar g6pc proteins. (DOCX 17 kb) [file 12864_2017_3727_MOESM5_ESM.docx]

|  | **Lamprey ENSPMAP00000002721** | **Gar *g6pca* ENSLOCP00000015000** | | **Gar *g6pcb* ENSLOCP00000015015** |
| --- | --- | --- | --- | --- |
| Coelacanth-ENSLACP00000022280 | 47.14 | | 54.26 | 67.33 |
| Tarsier-ENSTSYP00000006032 | 41.71 | | 50.85 | 58.81 |
| Wallaby-ENSMEUP00000005806 | 48.00 | | 57.39 | 64.20 |
| Guinea-ENSCPOP00000012071 | 48.29 | | 59.26 | 66.38 |
| Lesser-ENSETEP00000015235 | 48.86 | | 58.24 | 65.34 |
| Rat-ENSRNOP00000028033 | 49.43 | | 56.82 | 66.19 |
| Sloth-ENSCHOP00000007211 | 38.49 | | 47.71 | 56.21 |
| Kangaroo-ENSDORP00000013534 | 48.14 | | 56.98 | 66.95 |
| Rabbit-ENSOCUP00000013761 | 49.43 | | 59.94 | 67.33 |
| Shrew-ENSSARP00000008079 | 44.12 | | 51.94 | 59.71 |
| Pika-ENSOPRP00000011589 | 49.43 | | 59.09 | 68.18 |
| Microbat-ENSMLUP00000003741 | 48.00 | | 59.09 | 67.05 |
| Elephant-ENSLAFP00000011894 | 48.86 | | 57.10 | 66.48 |
| Hyrax-ENSPCAP00000004211 | 47.22 | | 54.44 | 67.22 |
| Gorilla-ENSGGOP00000027181 | 43.80 | | 52.44 | 61.89 |
| Marmoset-ENSCJAP00000024052 | 46.86 | | 56.53 | 67.05 |
| Gibbon-ENSNLEP00000014434 | 47.71 | | 57.39 | 67.05 |
| VervetAGM-ENSCSAP00000014681 | 48.29 | | 57.67 | 67.05 |
| baboon-ENSPANP00000020738 | 48.29 | | 57.67 | 66.76 |
| Macaque-ENSMMUP00000010594 | 48.29 | | 57.95 | 67.05 |
| Chimpanzee-ENSPTRP00000043906 | 46.73 | | 56.80 | 67.16 |
| Human-ENSP00000253801 | 48.00 | | 57.10 | 66.76 |
| Squirrel-ENSSTOP00000016360 | 49.14 | | 57.95 | 67.33 |
| Megabat-ENSPVAP00000008317 | 48.57 | | 58.81 | 68.18 |
| Hedgehog-ENSEEUP00000002301 | 48.63 | | 55.74 | 65.03 |
| Mouse-ENSMUSP00000019469 | 42.57 | | 52.84 | 61.36 |
| Horse-ENSECAP00000000378 | 49.14 | | 58.52 | 67.61 |
| Bushbaby-ENSOGAP00000008443 | 50.00 | | 59.09 | 67.61 |
| Panda-ENSAMEP00000000243 | 48.86 | | 57.95 | 67.61 |
| Ferret-ENSMPUP00000009890 | 49.14 | | 58.81 | 68.47 |
| Cat-ENSFCAP00000012186 | 48.00 | | 58.24 | 67.61 |
| Dog-ENSCAFP00000021606 | 47.71 | | 58.52 | 67.33 |
| Dolphin-ENSTTRP00000011131 | 46.55 | | 55.14 | 64.57 |
| Alpaca-ENSVPAP00000002373 | 48.33 | | 59.92 | 67.36 |
| Anole-ENSACAG00000029477 | 49.14 | | 58.81 | 67.61 |
| Cow-ENSBTAP00000013436 | 49.14 | | 58.81 | 67.61 |
| Sheep-ENSOARP00000004336 | 48.86 | | 58.81 | 68.18 |
| Xenopus-ENSXETP00000029871 | 50.14 | | 58.40 | 65.24 |
| Xenopus-ENSXETP00000053021 | 48.71 | | 55.56 | 66.95 |
| turtle-ENSPSIP00000005277 | 50.00 | | 60.51 | 69.03 |
| Anole-ENSACAP00000013381 | 51.14 | | 61.65 | 69.89 |
| Turkey-ENSMGAP00000004012 | 50.57 | | 61.36 | 69.60 |
| Flycatcher-ENSFALP00000000774 | 50.00 | | 58.92 | 64.87 |
| Finch-ENSTGUP00000002909 | 50.57 | | 59.38 | 65.62 |
| Finch-ENSTGUP00000002917 | 51.43 | | 58.81 | 65.34 |
