# Supplementary material for: Evolutionary history of glucose-6-phosphatase encoding genes in vertebrate lineages: towards a better understanding of the functions of multiple duplicates
Source: BMC Genomics. 2017 May 2;18:342. doi: 10.1186/s12864-017-3727-1 (PMC5414149; doi:10.1186/s12864-017-3727-1)
Supplement: Supplementary file 6 — Formulation and proximate composition of the two experimental diets used (NoCHO and HighCHO diets) in this experiment. (DOCX 19 kb) [file 12864_2017_3727_MOESM6_ESM.docx]

**Table S1.** Formulation and proximate composition of the two experimental diets used (NoCHO and HighCHO diets) in this experiment.

**Diets**

**NoCHO HighCHO**

Ingredients %

Fish meal^1^ 90.85 55.9

Fish oil^2^ 5.15 10.1

Starch^3^ 0 30

Vitamin mix^4^ 1 1

Mineral mix^5^ 1 1

Alginate^6^ 2 2

Proximate composition

Dry matter (DM, % diet) 97.03 96.34

Crude protein (% DM) 61.22 39.10

Crude lipid (% DM) 18.00 15.32

Gross energy (kJ/g DM) 22.06 21.64

Ash (% DM) 17.69 11.52

Carbohydrates (% DM) <1.0 28.28

^1^ Sopropeche, Boulogne-sur-Mer, France

^2^ fish oil; Sopropeche, Boulogne-sur-Mer, France.

^3^ Gelatinized corn starch (Roquette, Lestrem, France)

^4^ Supplied the following (kg^-1^ diet): DL-a tocopherol acetate 60 IU, sodium menadione bisulphate 5 mg, retinyl acetate 15000 IU, DLcholecalciferol 3000 IU, thiamin 15 mg, riboflavin 30 mg, pyridoxine 15 mg, vit. B_12_ 0.05 mg, nicotinic acid 175 mg, folic acid 500 mg, inositol 1000 mg, biotin 2.5 mg, calcium panthotenate 50 mg, choline chloride 2000 mg.

^5^ Supplied the following (kg^-1^ diet): calcium carbonate (40% Ca) 2.15 g, magnesium oxide (60% Mg) 1.24 g, ferric citrate 0.2 g, potassium iodide (75% I) 0.4 mg, zinc sulphate (36% Zn) 0.4 g, copper sulphate (25% Cu) 0.3 g, manganese sulphate (33% Mn) 0.3 g, dibasic calcium phosphate (20% Ca, 18% P) 5 g, cobalt sulphate 2 mg, sodium selenite (30% Se) 3 mg, potassium chloride 0.9 g, Sodium chloride 0.4 g.

^6^ Louis François, Marne-la-Vallée, France
