# Supplementary material for: Evolutionary history of glucose-6-phosphatase encoding genes in vertebrate lineages: towards a better understanding of the functions of multiple duplicates
Source: BMC Genomics. 2017 May 2;18:342. doi: 10.1186/s12864-017-3727-1 (PMC5414149; doi:10.1186/s12864-017-3727-1)
Supplement: Supplementary file 3 — Relative expression of g6pc genes in all species available in PhyloFish database (PDF 467 kb) [file 12864_2017_3727_MOESM3_ESM.pdf]

g6pca		Br	M	Gi	He	Int	Li	Kid	Bo	Ov	T	E
	Bowfin	8	6	1	28	4	100	1	2	0	6	nd
	Gar	1	0	0	9	45	100	46	1	0	0	8
	European eel	0	0	0	0	5	100	0	0	0	0	2
	Allis shad	NF	NF	NF	NF	NF	NF	NF	NF	NF	NF	NF
	Zebrafish	0	0	0	2	14	100	2	2	0	5	6
	Panga	0	1	0	1	0	100	4	2	0	0	50
	Black ghost	0	0	0	0	0	100	1	0	0	0	0
	Cave Mexican tetra	1	0	7	1	4	3	100	1	2	54	2
	Surface Mexican tetra	0	0	0	0	0	100	0	0	0	0	5
	Northern pike	0	0	0	0	1	100	0	1	0	0	4
	Eastern mudminnow	0	0	0	6	1	100	0	0	0	0	0
	European whitefish	0	0	0	0	0	100	0	1	0	0	3
	Brown trout	0	0	0	0	0	100	0	0	0	0	1
	Rainbow trout	0	0	0	0	0	100	0	3	0	0	0
	Brook trout	0	0	0	0	0	100	0	0	0	0	0
	Atlantic cod	NF	NF	NF	NF	NF	NF	NF	NF	NF	NF	NF
	Medaka	0	0	0	0	18	100	3	0	0	0	0

g6pcb		Br	M	Gi	He	Int	Li	Kid	Bo	Ov	T	E
	Bowfin	0	1	0	64	85	100	9	0	0	0	nd
	Gar	1	0	0	100	2	6	5	0	0	0	9

g6pcb1			Br	M	Gi	He	Int	Li	Kid	Bo	Ov	T	E
	European eel		0	0	0	1	4	100	0	0	0	0	2
	Allis shad		0	0	0	1	12	100	7	9	0	1	27
	Zebrafish		0	3	0	1	86	93	100	59	0	0	8
	Panga		1	0	1	0	1	100	37	2	1	1	6
	Black ghost		0	0	0	0	2	100	4	0	0	0	0
	Cave Mexican tetra		0	0	1	0	8	0	34	1	100	2	0
	Surface Mexican tetra		3	1	3	6	100	44	48	2	0	4	4
	Northern pike		0	0	0	0	7	100	0	7	0	0	4
	Eastern mudminnow		6	1	7	5	37	100	7	9	12	9	0
	European whitefish	g6pcb1a	2	0	1	1	37	100	1	2	0	0	1
		g6pcb1b	NF	NF	NF	NF	NF	NF	NF	NF	NF	NF	NF
	Brown trout	g6pcb1a	0	0	0	0	1	100	0	0	0	0	6
		g6pcb1b	0	0	0	0	0	100	0	0	0	0	2
	Rainbow trout	g6pcb1a	0	0	0	0	0	100	0	2	0	nd	nd
		g6pcb1b	2	1	0	1	17	100	1	1	0	nd	nd
	Brook trout	g6pcb1a	0	0	0	0	0	100	0	0	0	0	0
		g6pcb1b	0	0	0	5	1	100	0	0	0	0	1
	Atlantic cod		0	0	0	0	5	100	2	0	0	0	24
	Medaka		nd	0	0	0	31	100	6	1	0	0	0

g6pcb2			Br	M	Gi	He	Int	Li	Kid	Bo	Ov	T	E
	European eel		NF	NF	NF	NF	NF	NF	NF	NF	NF	NF	NF
	Allis shad		NF	NF	NF	NF	NF	NF	NF	NF	NF	NF	NF
	Zebrafish		0	0	0	100	0	0	0	0	0	0	0
	Panga		NF	NF	NF	NF	NF	NF	NF	NF	NF	NF	NF
	Black ghost		NF	NF	NF	NF	NF	NF	NF	NF	NF	NF	NF
	Cave Mexican tetra		0	0	1	100	0	33	1	0	9	13	0
	Surface Mexican tetra		0	1	2	100	1	0	9	1	2	37	1
	Northern pike		2	0	0	100	1	11	0	0	0	1	1
	Eastern mudminnow		NF	NF	NF	NF	NF	NF	NF	NF	NF	NF	NF
	European whitefish	g6pcb2a	NF	NF	NF	NF	NF	NF	NF	NF	NF	NF	NF
		g6pcb2b	NF	NF	NF	NF	NF	NF	NF	NF	NF	NF	NF
	Brown trout	g6pcb2a	24	2	5	100	3	2	8	6	1	4	9
		g6pcb2b	NF	NF	NF	NF	NF	NF	NF	NF	NF	NF	NF
	Rainbow trout	g6pcb2a	93	19	16	100	11	5	17	11	25	nd	nd
		g6pcb2b	100	9	9	97	8	4	18	9	5	nd	nd
	Brook trout	g6pcb2a	0	0	1	100	2	4	0	0	12	0	1
		g6pcb2b	NF	NF	NF	NF	NF	NF	NF	NF	NF	NF	NF
